# Regulation of cell-to-cell variability in divergent gene expression

**DOI:** 10.1038/ncomms11099

**Published:** 2016-03-24

**Authors:** Chao Yan, Shuyang Wu, Christopher Pocetti, Lu Bai

**Affiliations:** 1 Department of Biochemistry and Molecular Biology, University Park, Pennsylvania 16802, USA; 2 Center for Eukaryotic Gene Regulation, University Park, Pennsylvania 16802, USA; 3 Department of Physics, The Pennsylvania State University, University Park, Pennsylvania 16802, USA

## Abstract

Cell-to-cell variability (noise) is an important feature of gene expression that impacts cell fitness and development. The regulatory mechanism of this variability is not fully understood. Here we investigate the effect on gene expression noise in divergent gene pairs (DGPs). We generated reporters driven by divergent promoters, rearranged their gene order, and probed their expressions using time-lapse fluorescence microscopy and single-molecule fluorescence *in situ* hybridization (smFISH). We show that two genes in a co-regulated DGP have higher expression covariance compared with the separate, tandem and convergent configurations, and this higher covariance is caused by more synchronized firing of the divergent transcriptions. For differentially regulated DGPs, the regulatory signal of one gene can stochastically ‘leak' to the other, causing increased gene expression noise. We propose that the DGPs' function in limiting or promoting gene expression noise may enhance or compromise cell fitness, providing an explanation for the conservation pattern of DGPs.

## Abstract (web summary)

Gene expression noise affects cell fitness and development. Here, Yan *et al*. show that co-regulated divergent gene pairs (DGPs) suppress uncorrelated gene expression noise due to more synchronized transcription firing, and differentially regulated DGPs enhance gene expression noise due to transcription leakage.

## Graphical abstract

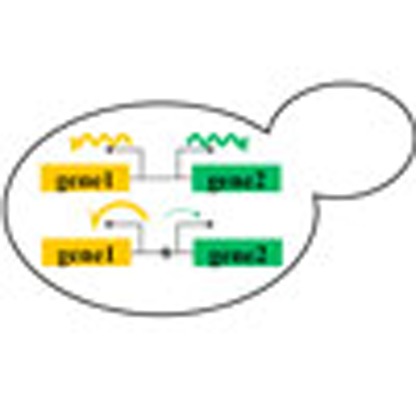

Cell proliferation and differentiation depend on rigorously controlled gene activities, yet gene expression is intrinsically variable. In recent years, large effort has been dedicated to characterizing the cell-to-cell variability, or ‘noise', of gene expression^1^,^2^,^3^,^4^. Large variations in the expression of house-keeping genes are likely to compromise cell fitness^5^,^6^,^7^; variations in some other genes can be beneficial by generating heterogeneous phenotypes in a population of genetically identical cells^8^,^9^,^10^,^11^,^12^,^13^,^14^,^15^. Given the physiological relevance, it is conceivable that cells have developed mechanisms to regulate gene expression noise. Indeed, recent studies have shown that noise can be modulated by promoter architecture, including the configuration of transcription factor binding sites, the sequence of TATA box and positioning of nucleosomes^6^,^8^,^16^,^17^,^18^. Beyond these *cis*-regulatory elements, a gene is embedded in a chromosomal context. The arrangement of neighbouring genes, also known as gene order, is thought to regulate the average level of gene expression^19^. However, the relationship between gene order and gene expression noise is unknown.

In this study, we focused on the noise regulation by divergent gene pairs (DGPs), that is, two neighbouring genes transcribed on opposite stands in a ‘head-to-head' configuration. About half of the whole yeast genome is organized in DGPs, most of which have short intergenic regions (between 200 and 800 bp)^20^. In fruit fly and human genomes, 32 and 10% of all genes form DGPs with intergenic distances <1 kb (refs 21, 22). Given the close proximity, a transcription factor associated with a DGP promoter may activate the two genes simultaneously, causing synchronized fluctuations in their expressions in individual cells (Fig. 1a,b). In other words, DGP may suppress the ‘uncorrelated expression noise' between the two genes. Such effect can be beneficial if the two gene products form a complex or function in the same pathway. In contrast, if the two genes in a DGP are differentially regulated by different transcription factors, there may be ‘crosstalk' between the two regulatory signals (Fig. 1c). If the ‘crosstalk' occurs stochastically and infrequently, it may result in enhanced variability of gene expression among genetically identical cells as well as in a single cell over time. The function of DGPs in regulating gene expression noise may also shape its conservation pattern through evolution. In particular, it may provide explanation to previous observations that the conservation of DGPs is positively correlated with co-expression^23^,^24^.

Here we tested these ideas by combining yeast genetics, single-cell gene expression assay and bioinformatics. We showed that two genes in a co-regulated DGP have higher covariance in their expression compared with other spatial configurations, and this higher covariance is caused by more synchronized firing of the divergent transcriptions. We also examined two differentially regulated DGPs and found that the regulatory signal of one gene can stochastically ‘leak' to the other, and in these two cases, the leakage causes increased cell-to-cell variability of gene expression. Finally, we proposed that the DGPs' function in limiting/promoting gene expression noise may enhance/compromise cell fitness, which is consistent with the observation that highly co-regulated DGPs are more conserved through evolution, and differentially regulated DGPs are less conserved.

## Results

### Probe *HTA2–HTB2* divergent promoter activity in single cells

To study the effect of co-regulated DGPs on gene expression covariance, we chose the yeast *HTA2–HTB2* gene pair as our model. *HTA2* and *HTB2* encode the highly conserved histones H2A and H2B. H2A and H2B function as a dimer *in vivo*, making it important to maintain their stoichiometry^25^,^26^. *HTA2–HTB2* promoter is cell cycle regulated^27^ and thus presents a stringent system for quantitative measurement of gene expression.

*HTA2–HTB2* promoter fires during a short window of time between G1/S and G2 (ref. 27). To evaluate its activity in single cells, we fused unstable Venus (a yellow fluorescent protein)^28^ to either side of the entire *HTA2–HTB2* intergenic region to generate *HTB2pr*-Venus and *HTA2pr*-Venus (same divergent promoter with different orientations) (Fig. 2a,b). We then integrated the reporters into yeast chromosome and monitored Venus intensity in live cells using time-lapse fluorescence microscopy over multiple cell generations (Fig. 2a,b; Methods)^6^,^29^,^30^,^31^. We also used Myo1-mCherry in these strains as a cell cycle marker^6^. These strains have intact endogenous *HTA2–HTB2* genes, and their growth rates are the same as wild-type cells.

Consistent with previous bulk measurements, Venus driven from either side of this promoter showed strong periodic changes in its intensity once every cell cycle (Fig. 2a,b). On average, *HTB2pr*-Venus and *HTA2pr*-Venus have identical cell cycle amplitudes, indicating that this divergent promoter has the same firing strength in both orientations (histograms in Fig. 2a,b; Methods). Note that in both cases, this amplitude varies up to four-fold among individual cell cycles. Such variation mostly reflects real changes in Venus expression since the measurement error accounts for <9% of the total fluctuations in the fluorescent intensity (Methods; Supplementary Fig. 1).

### Co-regulated DGPs have higher expression covariance

The experiment above monitored the *HTA2* and *HTB2* promoter activity individually. We next investigated the covariance of their activities in single cells by constructing a strain containing this promoter driving GFP and Venus as a DGP (Fig. 2c, Methods). For comparison, we separated *HTA2pr*-Venus and *HTB2pr*-GFP by keeping *HTB2pr*-GFP at the original locus and inserting *HTA2pr*-Venus into another chromosome (each with full-length *HTA2–HTB2* promoter; Fig. 2d). GFP and Venus have similar brightness and maturation rate in yeast, allowing us to directly compare their intensity after crosstalk elimination (Methods; Supplementary Fig. 2). The average Venus expression in these two strains are very similar (Supplementary Fig. 3), indicating that there is no position-dependent gene expression bias between these two chromosomal loci.

In both strains, the amplitudes of GFP and Venus expression in individual cell cycles are positively correlated (Fig. 2c,d). Such positive correlation is expected because several global factors, such as cell size and factor concentration, would have similar impact on both reporter genes in the same cell. Importantly, the covariance between GFP and Venus is significantly higher when divergently transcribed (Pearson correlation: *R*=0.65 versus 0.45, *P* value=0.0062; Fig. 2c,d; Methods), supporting a role of DGP in coordinating the expression of two genes in single cells. These *R* values are highly reproducible in three independent measurements.

**Figure 1:**
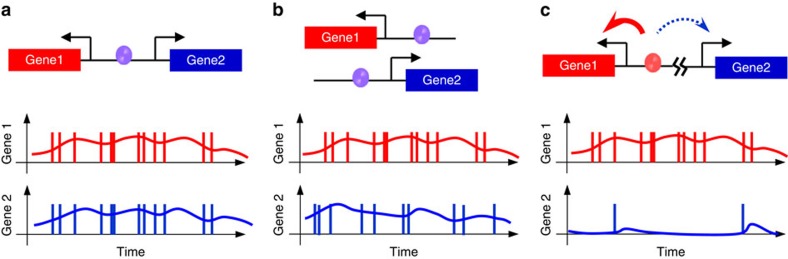
Models of the stochastic DGP transcription. (**a**) Two genes in a DGP may have enhanced covariance in their expression. Stochastic binding of transcription factors contributes to gene expression noise. In the case of a co-regulated DGP, where the two genes are under the control of the same transcription factor binding events, the transcription initiation of the two genes may occur simultaneously (vertical bars in the lower panel). Although the synchrony level of gene expression is likely to be reduced by subsequent stochastic events such as transcription elongation and termination, we still expect more synchronized fluctuations in the protein level of the two genes (curves in the lower panel). (**b**) Two separate genes driven by the same divergent promoter as in **a** may experience independent factor binding and asynchronized transcription firing. (**c**) In a differentially regulated DGP where the two genes respond to different transcription factors, the regulatory signal may ‘leak' stochastically to the wrong side. For every activation event of gene 1, if the leakage occurs sporadically in a fraction of cells, it may result in enhanced expression noise of gene 2. The notations in the diagrams are: black arrows, TSS; purple and red ovals, transcription factors; red and blue arrow in **c**, major and minor activation. The same notation applies to the following figures if not specified.

**Figure 2:**
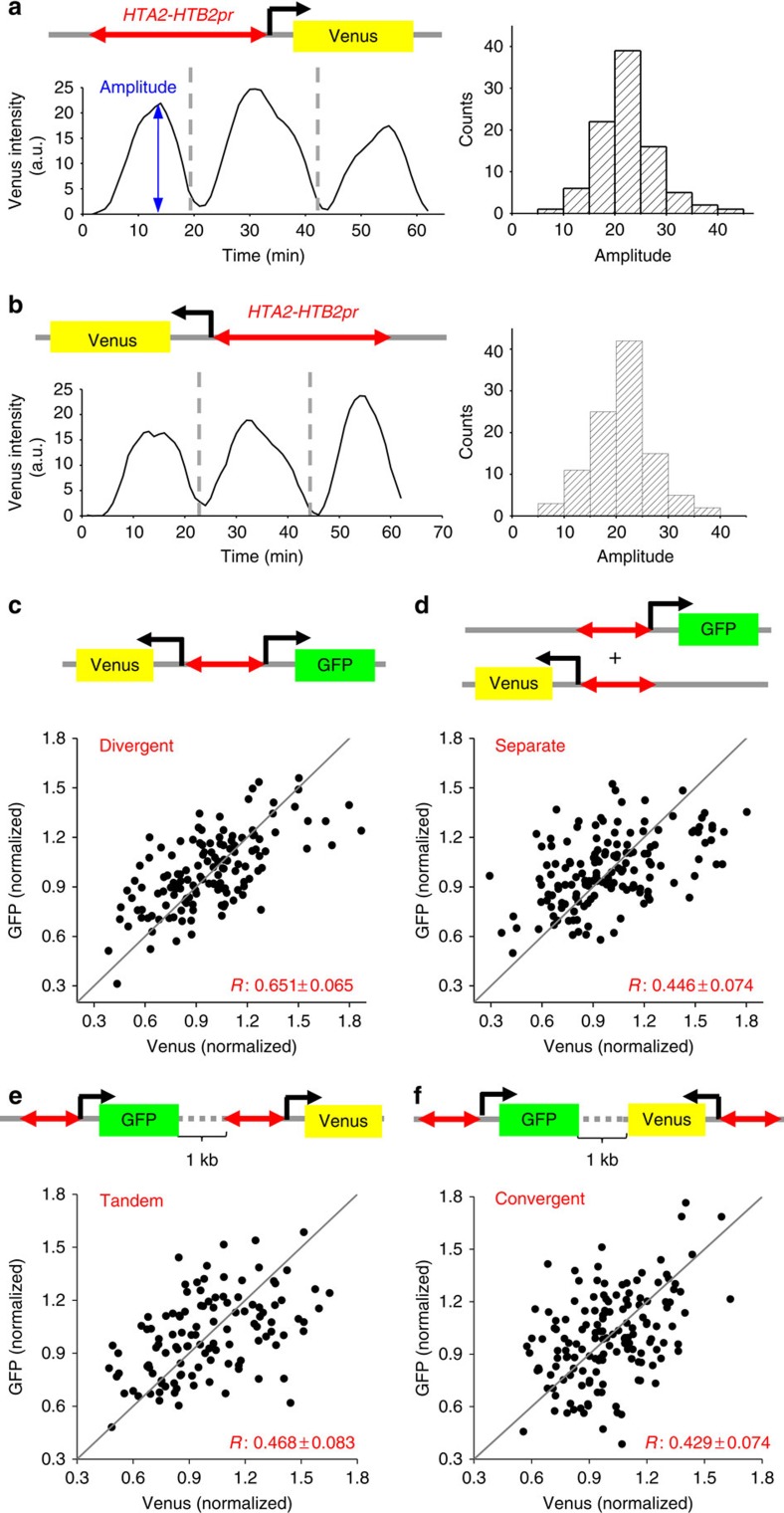
*HTA2–HTB2* DGP enhances expression covariance. (**a**,**b**) Characterization of the cell cycle regulated activity of the *HTA2–HTB2* promoter. The traces represent the Venus intensity as a function of time driven by this promoter from either orientation. The grey vertical lines indicate the time of cell division. The histograms of the cell cycle amplitudes, calculated as the peak-to-trough difference in the Venus signal per cell cycle, are also shown. a.u.: arbitrary unit. (**c**–**f**) Covariance between the GFP and Venus expression driven by the *HTA2–HTB2* promoter in divergent (**c**), separate (**d**), tandem (**e**) or convergent (**f**) configurations. The *x* and *y* axis for each dot in these plots correspond to the normalized Venus and GFP amplitudes in the same cell cycle. The *R* values are Pearson correlation coefficient (mean±s.e.m.; see Methods). Number of cell cycles: divergent: 140; separate 149; tandem: 115; convergent: 150.

The higher covariance of divergent GFP and Venus expression may be due to synchronized transcription firings (as hypothesized in Fig. 1) or simply due to their chromosomal proximity. In the latter scenario, two genes at the same locus have similar nuclear surroundings, which may contribute to their co-regulation. To differentiate between these two possibilities, we inserted the two reporters into neighbouring loci in tandem or convergent configurations (Fig. 2e,f). GFP and Venus expression in these strains showed low covariance (*R*=0.47 and 0.43), comparable to the case where the two reporters are separated on different chromosomes (Fig. 2d). Therefore, co-regulation was not enhanced by the spatial proximity *per se* in our system.

### Co-regulated DGPs show more synchronized transcription

**Figure 3:**
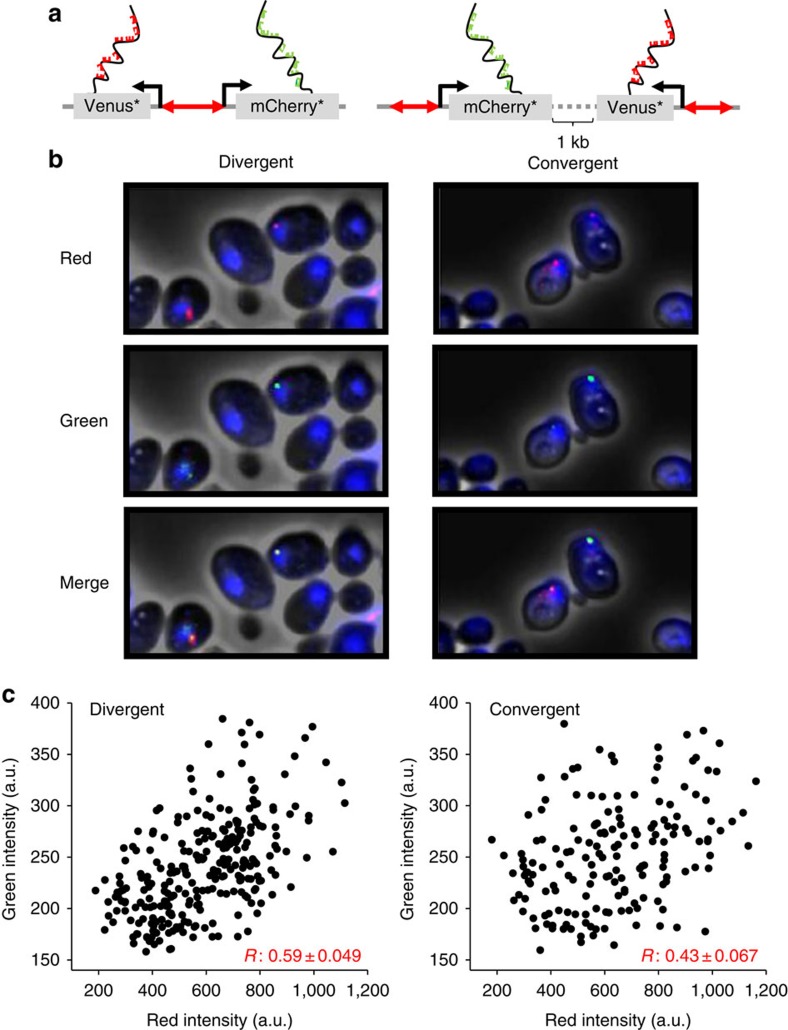
smFISH detected higher correlation in divergent transcription. (**a**) Cartoons illustrating the smFISH method for probing the divergently (left) or convergently transcribed (right) Venus* and mCherry* genes. The black curves represent nascent mRNAs at the transcription site (TS). The red and green blocks represent the CAL Fluor Red 610-labelled probes for Venus* and FAM-labelled probes for mCherry* transcript, respectively. (**b**) Typical FISH images of Venus* and mCherry* transcripts driven by *HTA2–HTB2* promoter in divergent (left) or convergent (right) configurations. Phase and DAPI channels merged with red, green or both fluorescent images were shown in the upper, middle or lower panels, respectively. (**c**) Correlation of the Venus* and mCherry* transcriptional activities. Each dot represents the fluorescent intensity of one TS in the red (*x*) and green (*y*) channels. The *R* values are Pearson correlation coefficient (mean±s.e.m.; see Methods). Number of TSs: divergent, 272; convergent, 185.

To probe the molecular origin of the higher DGP expression covariance, we used single molecule fluorescence *in situ* hybridization (smFISH) to directly visualize the nascent transcripts driven by *HTA2–HTB2* promoter in divergent and convergent configurations (Methods). We started from the genetic constructs in Fig. 2c,f, introduced a frame-shift mutation into Venus (Venus*) to avoid the interference from its fluorescent signal, and replaced GFP with a frame-shifted mCherry gene (mCherry*) so that its sequence can be differentiated from that of Venus* (Fig. 3a). After hybridization with a mixture of two differentially labelled probe sets targeting Venus* and mCherry* transcripts, we detected individual fluorescent ‘particles' that were only visible in the presence of target mRNAs (Supplementary Fig. 4; Methods). More than 90% of the particle-containing cells were either unbudded or small-budded, consistent with the G1/S activation of the *HTA2–HTB2* promoter (Supplementary Fig. 5; Methods). These observations confirmed the specificity of our smFISH signals.

For cells containing both Venus* and mCherry* reporters, the brightest red and green fluorescent particles often co-localize inside the nucleus (Fig. 3a,b). These particles represent the clusters of nascent Venus* and mCherry* mRNAs at the transcription sites (TSs)^32^,^33^. For every cell with detected TS (Venus* and/or mCherry*), we evaluated the fluorescent intensity at the TS in both red and green channels (Methods). In both divergent and convergent strains, the two fluorescent intensities fluctuate significantly relative to each other (Fig. 3c). These fluctuations largely reflect real differences in the transcriptional activities since the measurement error is much smaller (Supplementary Fig. 6). The covariance between the divergent transcription signals (*R*=0.59) is again significantly higher than the convergent ones (*R*=0.43; *P* value=0.0116; Methods) (Fig. 3c), indicating that the divergent genes are fired in a more synchronized fashion. These observations are consistent with a previous FISH experiment showing that the expression correlation between *GAL1* and *GAL10* (a DGP) is higher than that between *GAL1* and *GAL7* (non-divergent genes closely located on the same chromosome). Overall, our data in Figs 2 and 3 support the model in Fig. 1a,b.

### Leakage expression in differentially regulated DGPs

Unlike *HTA2–HTB2*, most DGPs in the genome are differentially regulated with two genes under the control of different transcription factors^34^. Our previous work showed that the regulation of two divergent genes in yeast can be ‘decoupled' by sequence-specific DNA-binding factors (blockage factors) that shield the proximal promoter from the action of more distant transcription regulators^34^. If this blockage mechanism is not 100% robust, there will be ‘leakage' of the regulatory signal from the upstream promoter, leading to undesired gene expression pattern (Fig. 1c).

**Figure 4:**
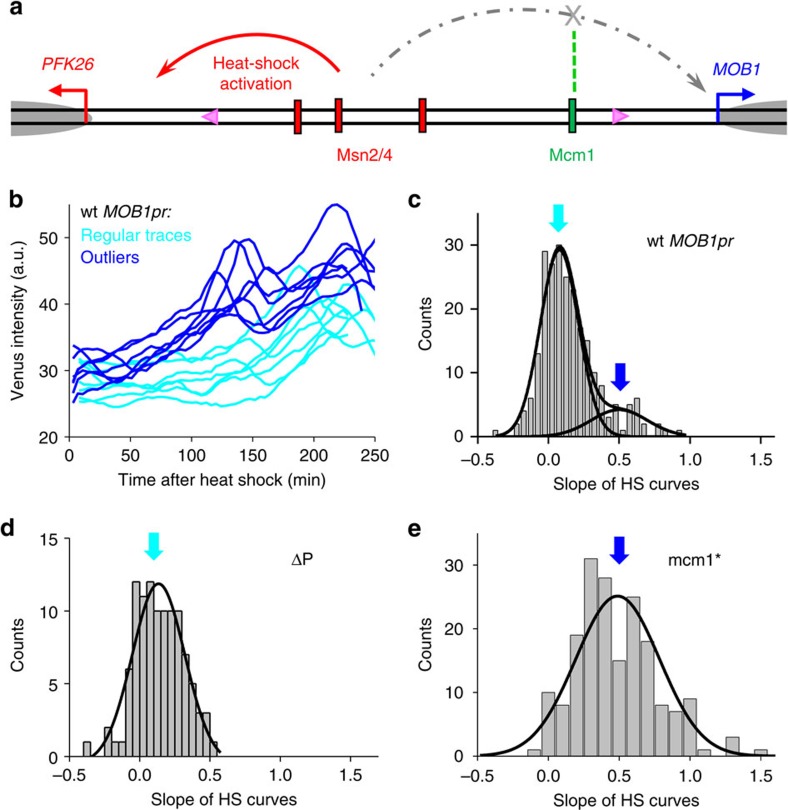
Stochastic *MOB1* heat-shock activation from the *PFK26*UAS. (**a**) Genomic structure of *PFK26*–*MOB1* divergent promoter. Red rectangles, putative Msn2/4 binding sites; green rectangle, Mcm1 binding site; pink triangles, two non-consensus TATA elements; red and blue arrows, TSSs and grey ovals, nucleosomes. (**b**) Representative heat-shock (HS) traces of Venus driven by *PFK26*–*MOB1* promoter from the *MOB1* side in single yeast cells. In most cells, *MOB1pr*-Venus showed no activation during the heat-shock phase (first 2 h), but started to oscillate when cells adapted and resumed growth (cyan traces). However, *MOB1pr* showed significant heat-shock response in a small fraction of cells (blue traces). (**c**-**e**) Histograms of heat-shock intensity driven by WT *MOB1* promoter (**c**) and its variants (**d**,**e**) among individual cells. This intensity was quantified by the slopes of the fluorescent traces during heat shock. ΔP, deletion of the *PFK26* side of the promoter including all the Msn2/4 binding sites; mcm1*, mutation of the Mcm1 binding site that leads to Mcm1 depletion from the promoter^34^. The WT *MOB1* promoter showed bimodal heat-shock response (**c**); in contrast, the two mutant promoters showed unimodal response corresponding to the lower (**d**) and higher peaks (**e**) respectively. Number of traces: WT, 236; ΔP, 105; mcm1*, 185.

To test this idea, we first examined a differentially regulated DGP, *PFK26*–*MOB1.* Under the heat-shock condition, the master stress regulator Msn2/4 binds and activates *PFK26pr* (Fig. 4a)^35^,^36^. The heat-shock activation of *MOB1*, however, is prevented by a Mcm1 protein associated downstream the Msn2/4 (ref. 34). To probe the potential leakage of the Msn2/4 signal, we measured *MOB1* heat-shock response in single cells (Methods). Interestingly, while *MOB1pr* has little heat-shock activity in most cells, mild activation of *MOB1pr*-Venus was detected in 15% of the population (Fig. 4b). Correspondingly, the slopes of these traces have a bimodal distribution that can be fit by two Gaussians (Fig. 4c; Methods). As a result, there is an increased cell-to-cell variability of *MOB1pr*-Venus expression during heat shock (Fig. 4b,c).

To understand the bimodality of *MOB1pr* heat-shock activity, we truncated the promoter sequence on the *PFK26* side (ΔP), eliminating all the potential Msn2/4 sites (Fig. 4d). Venus driven by the ΔP promoter exhibited close-to-zero heat-shock responses in all cells (Fig. 4d), indicating that Msn2/4 was responsible for the stochastic activation. In contrast, when we mutated the binding site of the blockage factor Mcm1 (mcm1*) in the full-length promoter, all cells showed positive heat-shock responses (Fig. 4e). The corresponding histogram of the heat-shock slopes had a unimodal distribution centered at ∼0.49, similar to the higher peak in that of the WT *MOB1pr* (centred at 0.5). These observations indicate that the heat-shock response of the WT *MOB1pr* is either fully ‘off' or fully ‘on' in individual cells, and the ‘on' cases may be due to the loss of Mcm1 blockage for extended period of time (see Discussion).

**Figure 5:**
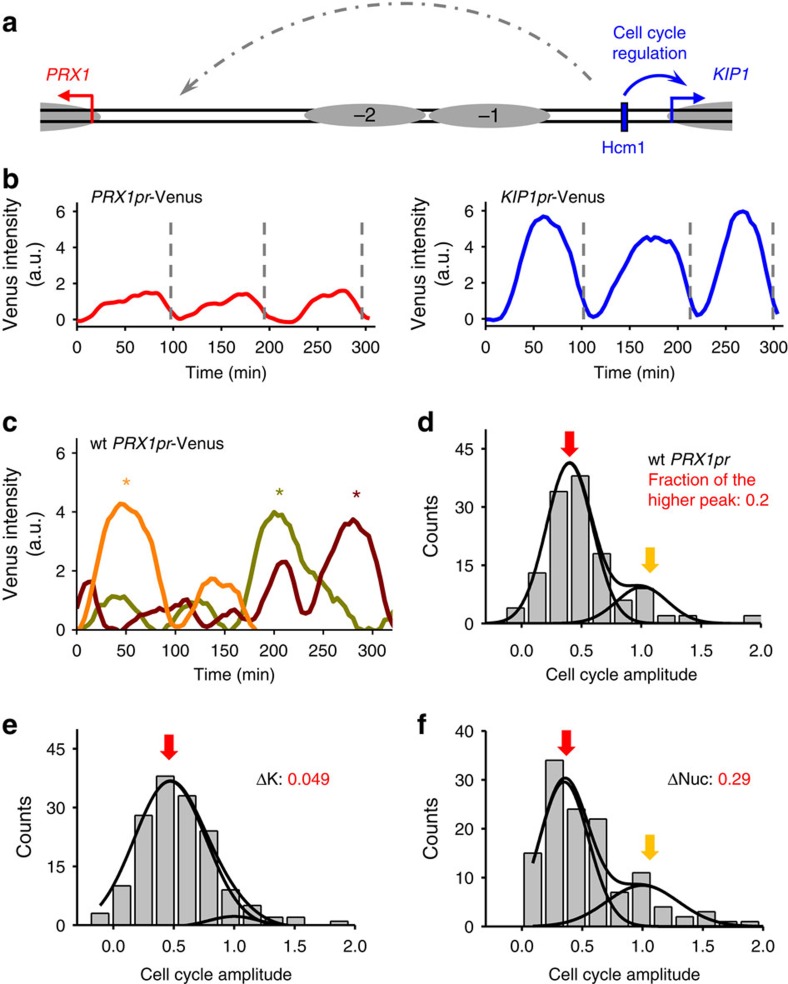
Stochastic *PRX1* cell cycle activation from the *KIP1* UAS. (**a**) Genomic structure of *PRX1–KIP1* divergent promoter. Blue rectangles, putative Hcm1 binding sites and grey ovals, nucleosomes. (**b**) Typical traces of Venus expression driven by WT *PRX1–KIPpr* from the *PRX1* orientation (left) or *KIP1* orientation (right) during vegetative growth. Each trace represents the Venus intensity as a function of time in a single yeast cell over multiple cell cycles. The vertical dash lines indicate the cell division time. In most cells, *KIP1pr*, but not *PRX1pr*, shows strong cell cycle-regulated activity. (**c**) Selected traces of *PRX1pr*-Venus with high cell cycle oscillation (marked by ‘*'). These traces occur in a small fraction of cells. (**d**-**f**) Histograms of the cell cycle amplitude of Venus driven by WT or mutant *PRX1* promoter. ΔK, deletion of the *KIP1* side of the promoter including nucleosome −1; ΔNuc, deletion of the nucleosomal sequences in the middle of the promoter. The WT *PRX1* promoter and ΔNuc showed bimodal cell cycle amplitude, and the higher peak is eliminated in the ΔK promoter. Number of cell cycles: WT, 129; ΔK, 155; ΔNuc, 125.

Similar stochastic crosstalk was observed on another differentially regulated DGP, *PRX1–KIP1* (Fig. 5a). *PRX1–KIP1* promoter has two separated nucleosome-depleted regions (Supplementary Fig. 7a)^37^, each containing the respective regulatory elements for *PRX1* or *KIP1*. A late S-phase transcription factor Hcm1 resides closer to the *KIP1* gene and drives the cell cycle regulation of *KIP1*, but not *PRX1* (refs 38, 39). Consistent with literature, we observed robust cell cycle regulation of *KIP1pr*-Venus with an average amplitude much higher than that of *PRX1pr*-Venus (Fig. 5b). Nevertheless, *PRX1pr*-Venus expression showed strong oscillations in a fraction of cell cycles (Fig. 5c), and the corresponding cell cycle amplitude had a bimodal distribution (Fig. 5d). The higher peak in this distribution disappeared when we deleted the *KIP1*-proximal sequences that contain the Hcm1 binding site (ΔK; Fig. 5e), indicating that the stochastic cell cycle regulation of *PRX1* originates from the leakage of Hcm1 activation. The mechanism underlying *PRX1* and *KIP1* differential regulation is still unknown. Deletion of an ∼300-bp nucleosomal sequence in the middle of the promoter joined the two nucleosome-depleted regions together (Supplementary Fig. 7b) and mildly increased the probability of high *PRX1* cell cycle regulation from 20 to 29% (ΔNuc; Fig. 5f). This result shows that the nucleosome separation (or simply the distance) only plays a minor role in decoupling the two genes.

### Highly co-regulated DGPs are more conserved in evolution

The effect on gene expression noise through divergent transcription likely plays a physiological role. For co-regulated DGPs, the maintenance of the stoichiometry of two subunits in a protein complex such as H2A and H2B may help to avoid the toxicity of the unpaired subunits. It may also facilitate the coordination of multiple proteins in the same reaction pathway. In contrast, differentially regulated DGPs cause leaky expression. Although large cell-to-cell variability in gene expression was shown to be beneficial for several environmental-responsive genes^8^,^9^,^10^,^11^, we suspect that in most cases (especially for house-keeping genes), sporadic gene expression triggered by unscheduled regulatory signal would have either neutral or detrimental effect on cell fitness. This idea is consistent with previous reports that the conservation of DGPs is positively correlated with their co-regulation^23^,^24^.

To confirm the conservation data, and in particular, to compare the conservation of DGP with the genome-wide baseline, we examined the change of neighbouring gene arrangement across the whole-genome duplication (WGD). WGD dramatically altered genome organization through duplication and deletion, which provided a unique opportunity to evaluate the evolution of gene order^40^. We expect gene orders that enhance/reduce cell fitness to be more/less conserved than the neutral selection.

**Figure 6:**
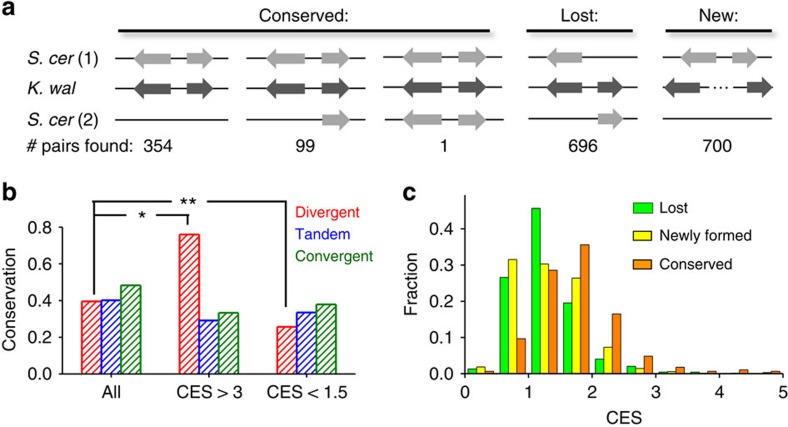
Evolution of DGPs across whole-genome duplication. (**a**) Definition of ‘conserved', ‘lost' and ‘new' DGPs by comparing the *S. cerevisiae* (post WGD) and *K. waltii* (pre-WGD) genome. (**b**) The conservation fraction for all divergent, convergent or tandem gene pairs, or the ones with CES>3 (highly co-regulated), or CES<1.5 (differentially regulated). Comparing with average, DGPs with high CESs are more likely to be conserved, and the ones with low CESs are less likely to be conserved. *P value<10^−2^; **P value<10^−3^. (**c**) The histogram of the CES for the conserved, lost and newly formed DGPs.

Based on the gene mapping in Kellis *et al*.^40^, we found 1,151 DGPs in *K. waltii* (pre-WGD) where both genes have orthologues in *S. cerevisiae* (post WGD). About 40% of these DGPs are conserved, and the rest are ‘lost' during evolution, that is, their orthologues are no longer in the DGP form in *S. cerevisiae* (Fig. 6a). There are also 700 newly formed DGPs in *S. cerevisiae*. This level of conservation is comparable to that of the tandem and convergent gene pairs (Fig. 6b). To understand how the conservation level is related to co-expression, we collected the co-expression score (CES) between two *S. cerevisiae* genes in the ‘conserved', ‘lost' and ‘newly formed' categories (Methods). Higher CES represents higher level of co-regulation. In agreement with previous reports^23^,^24^, highly co-regulated DGPs (CES>3) are more conserved than average (*P* value<10^−2^; Pearson's Chi-square test), and this trend was not observed for tandem and convergent gene pairs (Fig. 6b). Importantly, the average CES of the conserved DGPs (1.66±0.03; mean±s.e.m.) is significantly higher than that of the newly formed DGPs (new: 1.28±0.02) (Fig. 6c), indicating that the high CES is not merely a consequence of the DGP configuration. Out of the 12 conserved DGPs with CES>3, 8 encode subunits of histone and ribosome (Supplementary Table 1), indicating that there is a motivation to maintain the stoichiometry of these proteins. In contrast, the differentially regulated DGPs (CES<1.5) are significantly less conserved than average (*P* value <10^−3^; Pearson's Chi-square test) (Fig. 6b). These observations support the idea that the high covariance of co-regulated DGPs is beneficial for cell fitness and therefore these DGPs tend to be conserved. In the opposite, crosstalk between differentially regulated DGPs may be harmful and therefore minimized by natural selection.

## Discussion

Some divergent gene pairs are highly conserved among eukaryotic species but their biological function is not clear. Since the two genes in a DGP potentially share the same upstream regulatory sequences, it is commonly thought that DGP leads to co-regulation of functionally related genes^41^. However, two genes do not need to be divergently transcribed to achieve co-regulation; they simply need to be regulated by the same transcription factors. Indeed, in the budding yeast genome, only 34 out of the 44,489 gene pairs with CESs greater than or equal to 3 are DGPs. Even though the probability of finding high CES among DGPs (0.026) is still one order of magnitude higher than the genome-wide average (0.0022), these numbers clearly show that divergent transcription is not necessary for achieving high correlation between two genes. Therefore, the functional advantage of maintaining co-regulated DGPs in the genome is not clear.

The CESs were calculated based on microarray data, which measured the average gene expression among millions of cells. The cell-to-cell variability of gene expression is therefore not captured by these data. For some genes that function in multi-subunit complexes, it is conceivable that their balance needs to be stringently maintained in individual cells at all times. By measuring gene expression in single cells, we found that organizing two genes into a DGP serves as a mechanism to suppress the uncorrelated expression variation. Notably, for ribosome biosynthesis genes, it was proposed that adjacent gene pairs in all orientations (tandem, divergent and convergent) contribute to their coordination^41^. This was not found in our study: the tandem or convergent dual reporter genes driven by the *HTA2–HTB2* promoter do not have higher expression correlation than the separate ones. Therefore in our case, physical proximity alone does not play a major role in co-regulation. Instead, the smFISH measurement supported the idea that the suppression of uncorrelated noise is caused by more synchronized firing of the divergent transcriptions. When combined with literature on histone gene regulation, these data are consistent with the following picture: *HTA2* and *HTB2* are both activated by a transcription factor, Spt10, and the divergent promoter contains at least four Spt10 recognition sites^42^. Because of its strong binding cooperativity^43^, Spt10 tends to occupy these sites in an all-or-none fashion, resulting in simultaneous activation and coordinated expression of *HTA2* and *HTB2*. It should be noted that, although there are many multi-subunit complexes in yeast, highly correlated DGPs are limited to a few complexes, including histone and ribosome (Supplementary Table 1). We suspect that this is because these complexes play such fundamental roles, and the expressions of their subunits need to be better balanced.

Besides coordinating the expression of two genes in single cells, co-regulated DGPs may also allow the co-evolution of their regulation. Mutation in the divergent promoter sequence, especially in the upstream activating sequence (UAS), can have a similar impact on the two genes. These changes can fine-tune the expression level of the two genes without affecting their stoichiometry. This potential advantage may also contribute to the conservation of co-regulated DGPs.

Most DGPs in the genome are differentially regulated. So far we have characterized gene expression noise in two such DGPs and found transcription leakage in both cases, suggesting this is a wide-spread phenomenon. The decoupling between *PFK26* and *MOB1* is due to sequence-specific ‘blockage factors'^34^, and we suspect that the differential regulation of *PRX1* and *KIP1* is based on a similar mechanism. The leakages of the *PFK26* and *KIP1* signals occur sporadically in a small fraction of cells, causing bimodal gene activation in WT *MOB1* and *PRX1* promoters (Fig. 4c,d). This may be due to infrequent association and dissociation of the blockage factors. Alternatively, it can be caused by a double negative feedback loop between the binding of blockage factors and the leakage transcription^44^,^45^: the factors prevent the transcription initiation in the wrong orientation, but once such transcription starts, the blockage factors can no longer bind. In fact, in our previous experiments with synthetic promoters, we found some evidence showing that a strong transcription can lead to the eviction of blockage factors^34^. This can explain why the leakage transcription is either fully ‘on' or fully ‘off' among individual cells (Fig. 4c,e). Cells can prevent these unintended transcripts through more blockage factors, longer intergenic regions or other decoupling mechanisms. The current configuration of the differentially regulated divergent genes may reflect a balance between the fitness cost of the leakage transcripts and the cost to achieve substantial noise suppression.

## Methods

### Strains and plasmids

Standard methods were used to construct the strains and plasmids. All strains are based on W303. All the ‘wild-type' promoters were constructed as the entire intergenic region between the two divergent open reading frames (ORFs). The divergent *HTA2–HTB2* promoter driving GFP and Venus were integrated into *CLN2* locus at chrXVI: 65106 (Fig. 2c). The strains in Fig. 2d–f were derived from a common strain that contains *HTB2pr* driving GFP inserted into the *CLN2* locus at chrXVI: 65106. *HTA2pr* driving Venus was then integrated with different configurations relative to *HTB2pr*: (1) at chrXVI: 64037 in a tail to tail orientation (convergent); (2) at chrXVI: 64037 in a tail to head orientation (tandem); (3) at the *TRP1* locus on ChrIV (separate). Construction of the strains containing *PFK26–MOB1* promoter driving reporters were described in our previous work^34^, and the strains with *PRX1–TRP1* promoters were constructed with similar methods.

### Time-lapse fluorescence microscopy and data analysis

For the time-lapse measurements, we placed live yeast cells under an agar pad made with growth media and recorded fluorescent images every 4 min over 5–10 h (refs 6, 34). The cell cycle and heat-shock expression data was collected under 30 °C and 37 °C, respectively. An objective heater was used to keep the sample surface temperature as desired (measured by Omega surface thermocouple). The cell cycle curves were smoothed and corrected by subtracting a baseline connecting flanking troughs, and the peak height in each cell cycle represents the cell cycle amplitude. The heat-shock response was quantified by the slopes of fluorescence trace during the first 2 h of heat shock before cells adapted to the higher temperature and resumed growth. S.e.'s of the Pearson correlation coefficient in Figs 2 and 3 were calculated as 
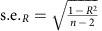
. To evaluate the significance between two independent correlation coefficients, each *R* value was first transformed to *Z* scores through Fisher *z*-transformation, and the pooled s.e. was calculated as 
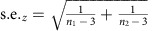
. *Z* score between the two *R* values was then calculated as 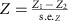 and then transformed to *P* value.

### Estimation of measurement error in single-cell microscopy

The measurement uncertainty most likely originates from the variations in the focusing position and the opening duration of the fluorescence shutter. To estimate the magnitude of this uncertainty, we repeated the time-lapse fluorescence measurement using the same cells with stage repositioning and re-focusing (Supplementary Fig. 1). The measurement error was calculated by the squared deviation between measurement 1 and 2.

### Two-colour data analysis

GFP and Venus have significantly overlapped spectrum, and for two-colour experiment, we eliminated the crosstalk by the following linear equation:


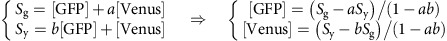


Where *S*_g_ and *S*_y_ are the signals we acquired in the GFP and YFP channel; [GFP] and [Venus] present the GFP and Venus concentration. The crosstalk parameters *a* and *b* were obtained using strains containing only GFP or Venus. The values of *a* and *b* depend on the filter, light source spectrum and the exposure settings in the GFP and YFP channel. With our current experimental condition (GFP filter: Chroma 49002, YFP filter: 49003, light source: OSRAM ARC Lamp, GFP exposure with 30% intensity for 0.1 s, YFP exposure with 55% intensity for 0.2 s), *a* and *b* equal to 0.409 and 1.036, respectively. See Supplementary Fig. 2 for fluorescent traces after crosstalk elimination. Importantly, the GFP peak value of the Venus-only strain was close to 0 after crosstalk elimination (0.056±0.052; mean±s.d. normalized by the average intensity of *HTB2pr*-GFP), and so is the Venus peak value in GFP-only strain (0.064±0.047) (normalized by the average intensity of *HTA2pr*-Venus). These numbers show that our method can effectively remove crosstalk.

### smFISH assay

We slightly modified a previously developed protocol of single molecule FISH^46^,^47^. We targeted the Venus* transcript with 28 CAL Fluor Red 610-labelled probes and mCherry* with 31 FAM-labelled probes (LGC Biosearch Technologies). After fixing the yeast cells with formaldehyde for 45 min at room temperature and permeating the membranes with 70% ethanol overnight at 4 °C, we performed hybridization in 50 μl solution overnight at 30 °C. The final concentration of each set of the probes is 150 nM. After DAPI staining, the cells were placed between a coverslip and a 1.5% agar pad made with 2 × SSC buffer, and imaged under a Leica DMI6000b fluorescent microscope. To detect three-dimensional FISH signals, the images were taken at seven focal planes (*z*-stack), 0.4 μm apart. The images were then analysed using MATLAB programs developed in our lab. After processing, the programs generated the cell and nucleus boundaries, as well as the locations of FISH dots with intensities above a threshold. The brightest dot inside the nucleus was assigned as the TS. For all detected TSs (either in the red or green channel, or both), we quantified the TS intensity within a 3 by 3 pixel area at the TS location for all seven *z* positions, and used the maximum across the *z*-stack as the final intensity.

### Nucleosome mapping

Nucleosome occupancy was measured by MNase digestion followed by stacking quantitative PCR. We first grew 10 ml cell to OD ∼0.15, collected the cell, and washed in 0.5 ml water. Then we re-suspended the cells in 0.5 ml of sphaeroplasting solution (1 M sorbitol, 0.5 mM 2-mercaptoethanol, 0.18 mg ml^−1^ zymolyase), incubated at room temperature for ∼5 min with gentle stir. We then collected the cell, wash in 1 ml of 1 M sorbitol, then re-suspend the pellet in 200 μl of digestion buffer (1 M sorbitol, 50 mM NaCl, 100 mM Tris-Cl (pH 7.4), 5 mM MgCl_2_, 1 mM CaCl_2_, 1 mM 2-mercaptoethanol, 0.5 mM spermidine, 0.075% NP-40, micrococcal nuclease with final concentration 1–10 unit ml^−1^) for ∼8 min in 37 °C. After terminating the MNase digestion by adding 20 μl quench buffer (250 mM EDTA, 5% SDS), we purified the ∼150 bp DNA, and proceeded with the qPCR analysis with stacking PCR primer pairs^48^. The PCR products were all ∼100 bp in length, and the distances between adjacent primers were typically ∼50 bp. We used the nucleosome −1 on the *PHO5pr* as the standard to scale the occupancy from 0 to 1.

### Bioinformatics analysis of DGP conservation

We developed MATLAB software for the statistical analysis. In both *S. cerevisiae* and *K. waltii*, we defined ‘DGPs' as head-to-head gene pairs with distance between the two ORFs <1,000 bp. We used the data in ref. 40 to map the homologous genes in these two species. Lacking CES data in *K. waltii*, we retrieved the CES in *S. cerevisiae* from Serial Pattern of Expression Levels Locator (SPELL)^49^.

## Additional information

**How to cite this article:** Yan, C. *et al*. Regulation of cell-to-cell variability in divergent gene expression. *Nat. Commun.* 7:11099 doi: 10.1038/ncomms11099 (2016).

## Supplementary Material

Supplementary Information Supplementary Figures 1-7, Supplementary Table 1 and Supplementary Reference
