# Supplementary material for: Regulation of cell-to-cell variability in divergent gene expression
Source: Nat Commun. 2016 Mar 24;7:11099. doi: 10.1038/ncomms11099 (PMC4820839; doi:10.1038/ncomms11099)
Supplement: Supplementary Information — Supplementary Figures 1-7, Supplementary Table 1 and Supplementary Reference [file ncomms11099-s1.pdf]

Supplementary Figures

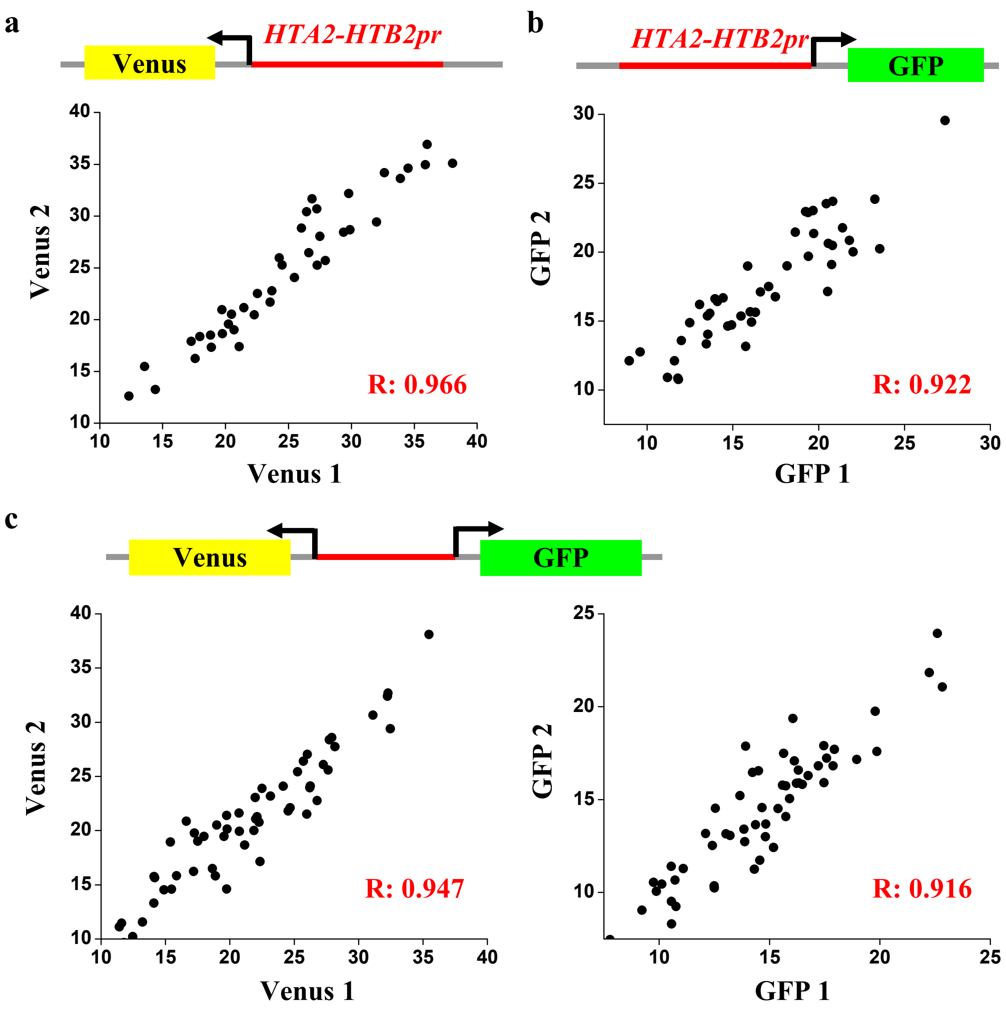

Supplementary Figure 1. Estimation of the measurement error in time-lapse microscopy. In strains expressing Venus, GFP, or both **(a-c)**, We repeated the fluorescent measurements on the same cells with stage repositioning and re-focusing. The x and y axis represent data from the two repeats. In all three cases, the cell-cycle amplitudes from different measurements were highly reproducible. The measurement uncertainties shown in these plots account for a few percent (4-9%) of the total variations in the cell cycle amplitudes in Fig. 2.

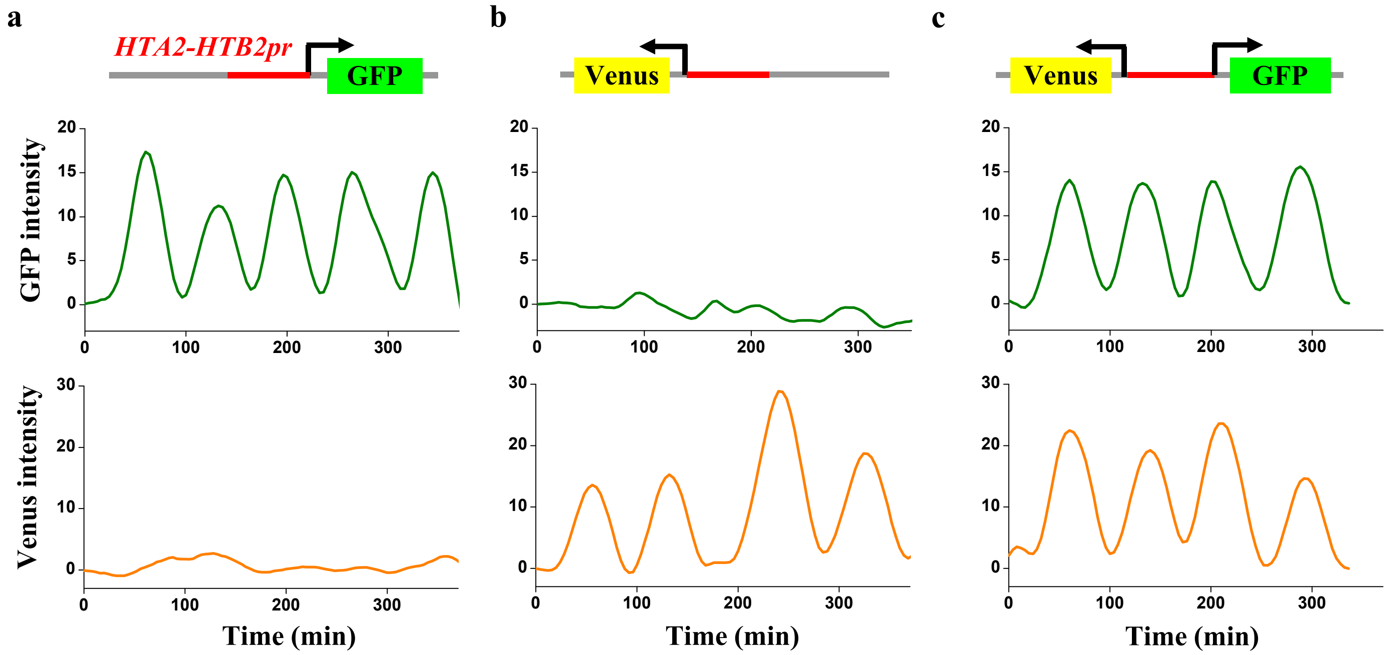

Supplementary Figure 2. GFP and Venus intensity after crosstalk elimination in three strains. The strain containing GFP but not Venus showed strong GFP signal and close-to-zero Venus signal **(a)**, indicating that the GFP leakage into the YFP channel was successfully eliminated, and vice versa **(b)**. The strain in **(c)** showed synchronized oscillation in GFP and Venus expression.

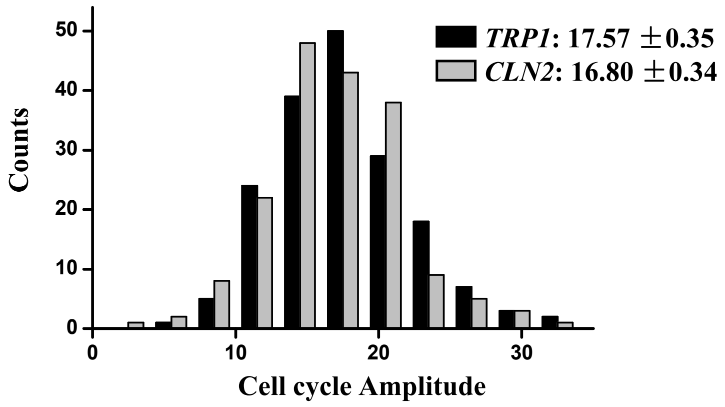

Supplementary Figure 3. Venus expression at the *TRP1* (black) or *CLN2* (grey) locus. The expression profile at these two loci are essentially the same.

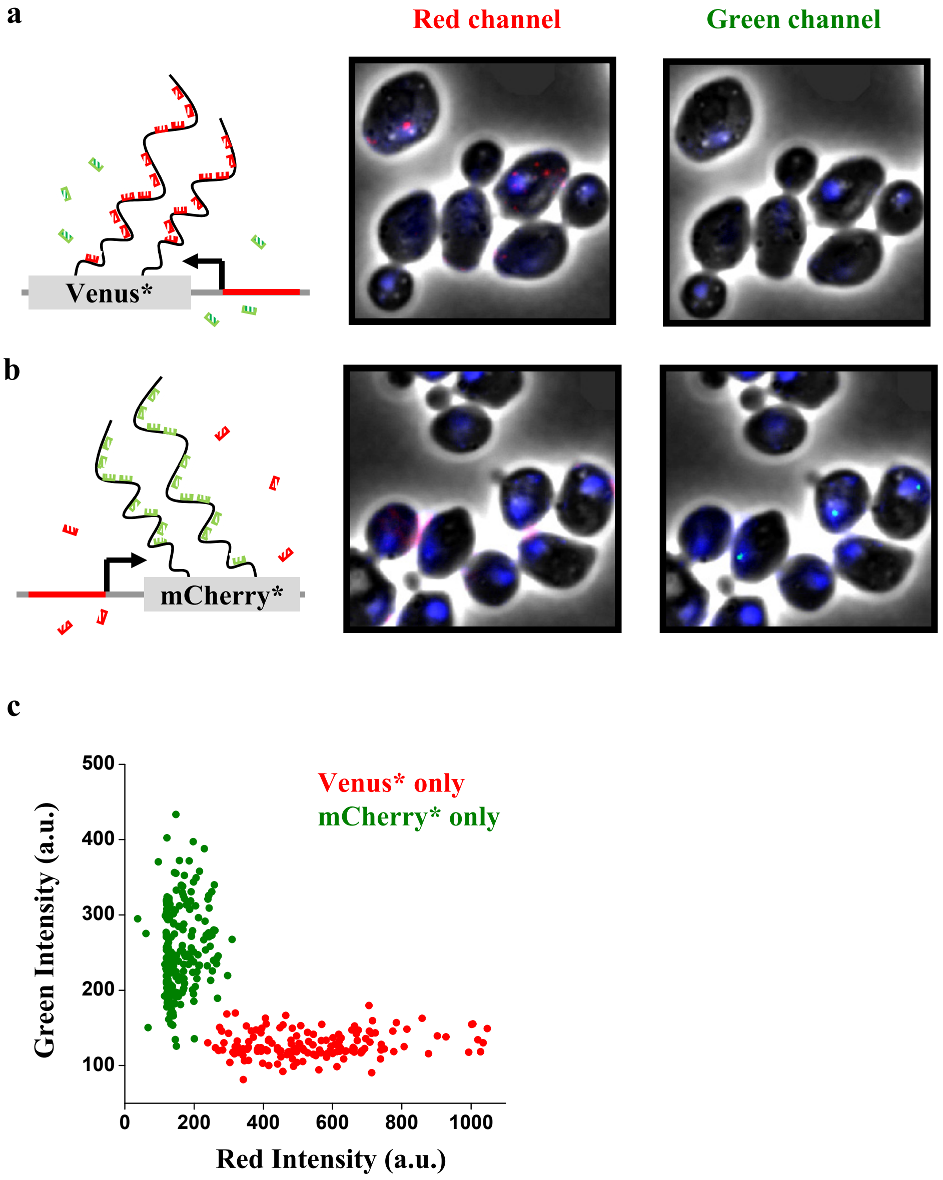

Supplementary Figure 4. Specificity of the smFISH probes. We mixed the Venus* and mCherry* probes together and hybridized to cells containing either *HTA2pr*-Venus* **(a)** or *HTB2pr*-mCherry* **(b)**, but not both. The middle panels are merged images from the phase, DAPI, and the red fluorescence channels; the right ones are from the phase, DAPI, and the green fluorescence channels. The FISH signals (red or green particles) were only detected in the presence of target mRNAs, showing that the FISH signal comes from specific probe-mRNA hybridization. **(c)** Quantification of the TS intensity. For every TS we detected, we plotted the fluorescence intensity at the TS location in both channels. The plot shows that the red and green signals are well decoupled.

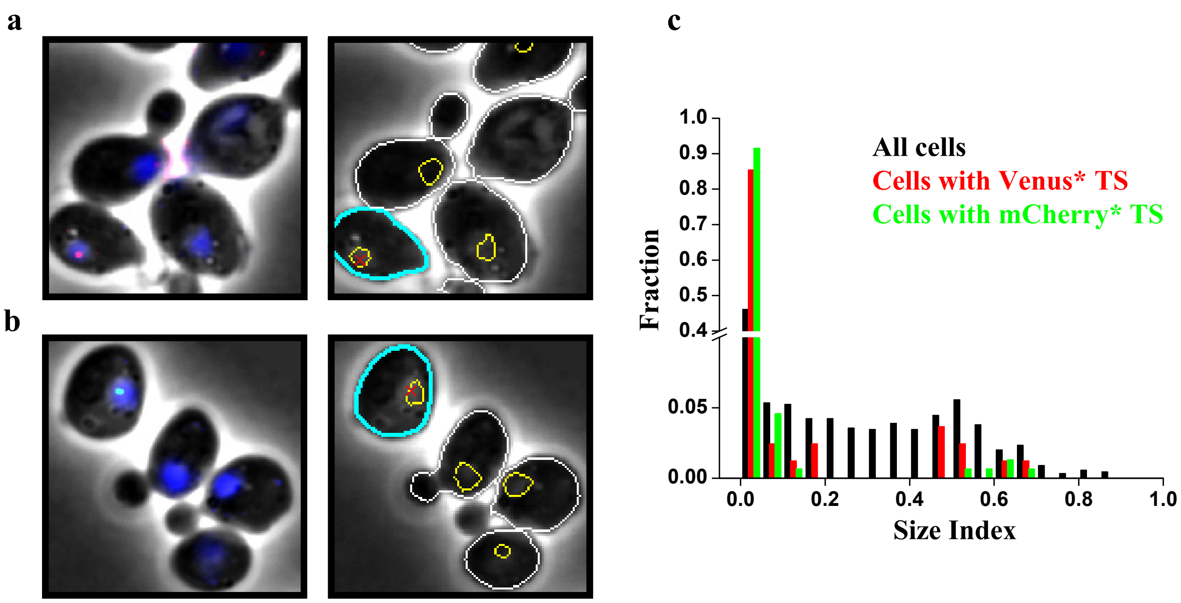

Supplementary Figure 5. Cell-cycle dependence of the smFISH signal. (a&b) smFISH images and analysis. Cells containing only *HTA2pr*-Venus* **(a)** or only *HTB2pr*-mCherry* **(b)** were hybridized to the corresponding probes. We developed a MATLAB program to analyze the cell and nuclei boundaries. The program also identified strong FISH signals within a nuclei, which likely reflected the nascent transcription sites (TS). Cells with TS were highlighted with cyan boundaries. **(c)** Venus* and mCherry* FISH signals tend to be detected in unbudded or small-budded cells. We use the size index (daughter cell area divided by the mother cell area) to estimate the cell cycle stage for each individual cell in the image. This index is 0 for G1 cells (unbudded), and gradually increases as cells go through the cell cycle. When we counted all cells (N=898), 46% were unbudded or small-budded (the first bin in the distribution); but for the cells where TSs were detected (N=82 and 153), this fraction became ~90%. This is consistent with the activation timing of the *HTA2-HTB2* promoter at the G1/S transition. This result further confirmed the specificity of the FISH signals. A small fraction of cells with TS have large size index. We think that this is due to errors in the cell cycle assignment (for example, it is sometimes hard to differentiate between cells at late mitosis, G1, or G1/S).

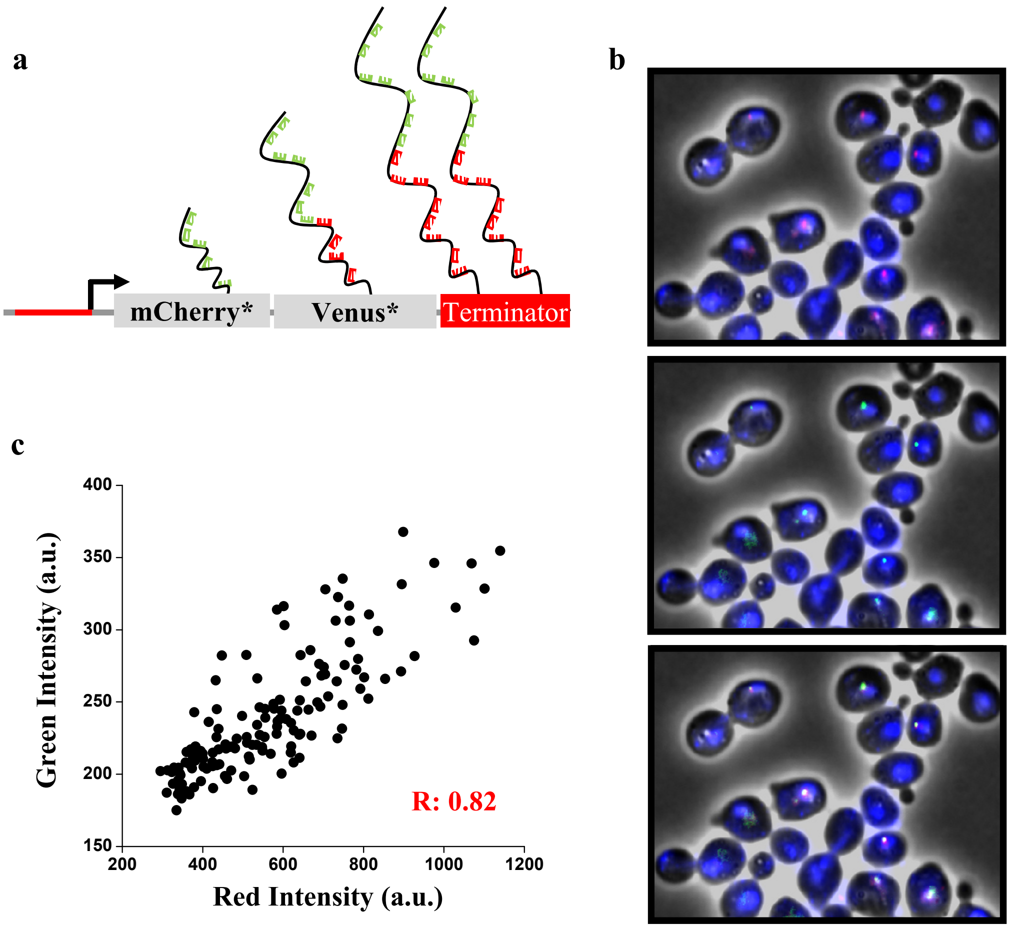

Supplementary Figure 6. Estimation of the smFISH measurement noise. (a) Construct of the smFISH measurement using fused mCherry*-Venus* mRNA. Since mCherry* and Venus* transcripts are short (712 and 718 bps), we reasoned that nascent mRNAs at the TS should accumulate at the termination site: if the elongation rate is ~40nt*s^{-1}, it takes 36s to elongate on the fused genes, while termination can take 70s according to a previous measurement[1]. Therefore, the TS intensity in the red and green channels should correlate well with each other. The fluctuation between these two intensities serves as a upper limit for the measurement error. **(b)** Typical FISH images. The three panels showed phase and DAPI channels merged with red, green, or both channels. **(c)** The correlation between the TS intensities in the red and green channels. The two intensities correlates well, with a correlation efficiency of 0.82.

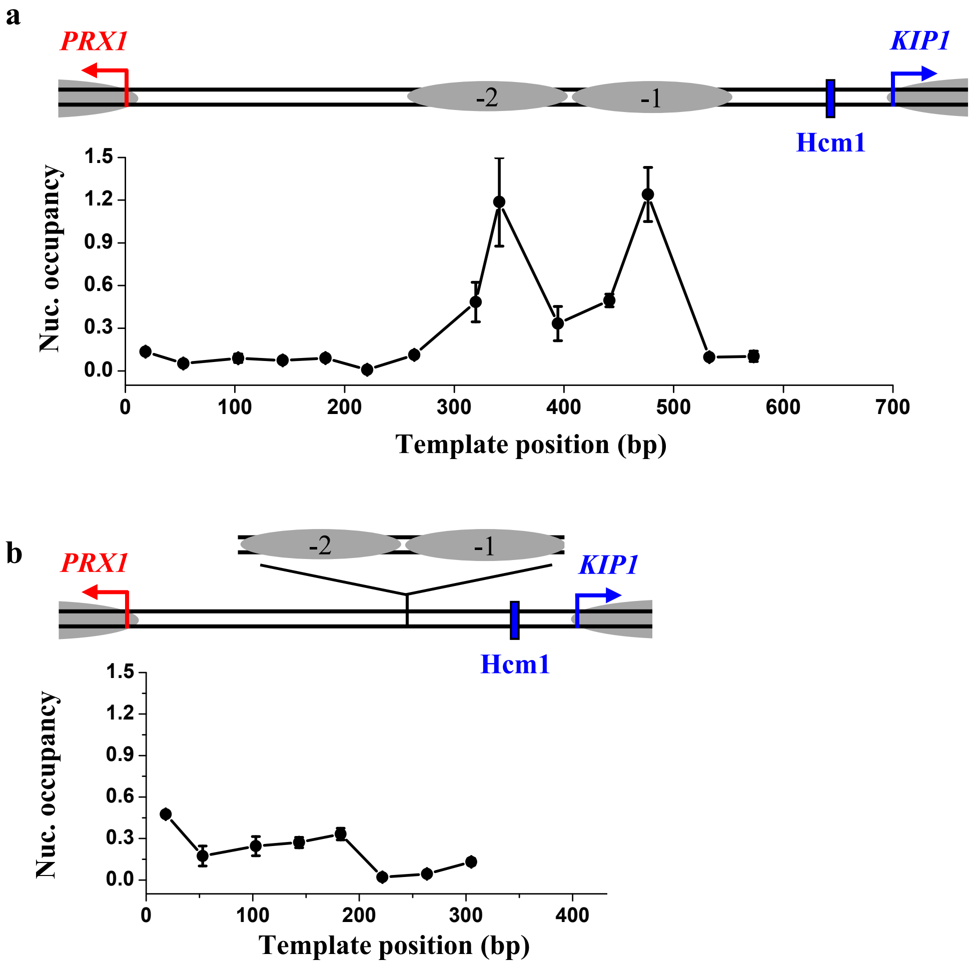

Supplementary Figure 7. Nucleosome distribution on the wt *PRX1-KIP1* promoter (a), and on the ΔNuc variant (b). The wt promoter has two separated nucleosome-depleted regions. After deletion of the nucleosome -2 and -1 sequence, the remaining *PRX1-KIP1* intergenic region became nucleosome-free. The position 0 in the x axis corresponds to the *PRX1* TSS. The error bars represent the s.e.m. of two biological replica.

Gene 1	Gene 2	CES	Category
HTB2	*HTA2*	4.7	Histones
HTA1	*HTB1*	4.4	
HHT1	*HHF1*	4.3	
HHF2	*HHT2*	4.1	
RPP2A	*RPS15*	4.4	Ribosome-related
NOP2	*GCD10*	3.7	
LCP5	*NSA2*	3.7	
SSF1	*RRP3*	3.6	
SNZ1	*SNO1*	4.1	Others
CMC1	*MRPL31*	3.3	
LTV1	*RPC25*	3.2	
BIO3	*BIO4*	3.2	

Supplementary Table 1. List of conserved DGPs with CES > 3. Many genes in these DGPs encode for subunits of histone and ribosome.

Supplementary Reference

1. Larson, D. R., Zenklusen, D., Wu, B., Chao, J. A., Singer, R. H. Real-time observation of transcription initiation and elongation on an endogenous yeast gene. *Science* **332**, 475-478, (2011).
